# A versatile genetic tool: haploid cells

**DOI:** 10.1186/s13287-017-0657-4

**Published:** 2017-09-29

**Authors:** Yanni Li, Ling Shuai

**Affiliations:** State Key Laboratory of Medicinal Chemical Biology, Nankai University, College of Pharmacy, Tianjin, 300350 China

**Keywords:** Haploid, Pluripotency, Diploidization, Genetic screening

## Abstract

Haploid cells are excellent tools to study gene function as they contain a single copy of the genome and are thus unable to mask the effect of mutations. Recently, haploid embryonic stem cells, which are capable of self-renewal and potentially differentiating into other cell types despite having only one set of chromosomes, have been established in several species. These unique haploid cells allow us to seek recessive gene functions in mammals, and have had a profound influence on the field of genetic screening and drug target identification. In this review, we briefly introduce advances and breakthroughs in haploid cell line research and broadly discuss the versatile application thereof.

## Background

In evolutionary terms, almost all cells in sexual organisms are diploid, with haploid cells, which cannot further divide, being restricted to gametes. Haploid cells in yeast and plants can show gene mutation phenotypes without any allelic backup, so are utilized extensively in genetic engineering [1]. Although haploid cells in animals have been explored since the 1970s, it was not until 2009 that the first vertebrate haploid embryonic stem cell (haESC) line was developed. This cell line was generated from Medaka fish embryos and introduced a brand new system for analyzing recessive phenotypes in vertebrates [2]. Two years later, mouse haESCs were established, thereby initiating genetic screening in mammals with haploid cells [3, 4]. After this, monkey [5] and rat [6] haESCs were respectively derived, raising the feasibility of using haploid cells in other mammalian species for genetic studies. Recently, human haESCs were achieved from chemically activated human eggs, thus facilitating the research of genetic diseases and gametogenesis in humans [7, 8]. Compared to previous near-haploid tumor cells [9], haESCs have intact genomes which lack mutations [10] and are pluripotent to three germ layers both in vitro and in vivo [11, 12]. Besides differentiation potential, haESCs have special reproductive functions like gametes, which yield transgene delivery from cells to animals via introcytoplasmic injection [12, 13]. What is more, haESCs have unlimited self-renewal abilities and are receptive to advanced gene editing methodologies. This allows these cells to generate homozygous genotypes containing only one set of chromosomes [14], thus making them a perfect tool to target gene functions associated with recessive traits.

In this review, the properties of haESCs and recent breakthroughs associated with their use in genetic screening are introduced. Key unsolved issues involving haESCs, as well as their future application in lineage specific gene function discovery, are raised.

### Identity of haploid cells

**Fig. 1:**
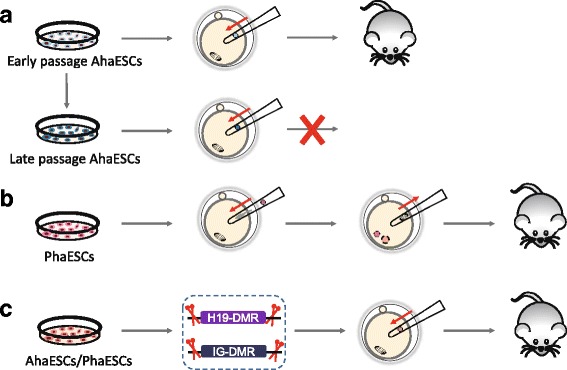
Strategies to generate offspring with haESCs. **a** Intracytoplasmic injection of androgenic haESCs (AhaESCs) in early and late passages. AhaESCs can produce live offspring only in early passages, because of the loss of paternal imprinting in long-term cell culture processes. This reduces developmental potential of AhaESCs. **b** By replacing the female pronucleus with parthenogenic haESCs (PhaESCs), the fertilized zygote can produce healthy progeny. **c** By knocking out the *H19*-DMRs and *IG*-DMRs of haESCs (AhaESCs and PhaESCs), genetically modified haESCs can efficiently produce mice via intracytoplasmic injection. DMR differentially methylated region, haESC haploid embryonic stem cell

haESCs express specific pluripotent genes and are able to form embryoid bodies and teratomas. They also share some common features with conventional diploid ESCs, such as colony morphology, unlimited self-renewal ability, and pluripotency. When injected into a blastocyst, mouse haESCs can contribute to the chimera through germline transmission [15]. Except for pluripotency, rodent haESCs are proven to be reproductive by replacing the genome with gametes. Intracytoplasmic injection of androgenic haESCs could support full-term development of the embryos in mice [12] and rats [6] (Fig. 1a). Additionally, substituting maternal pronuclei of zygotes with parthenogenic haESCs was shown to result in the birth of mice that retained maternal genetic and epigenetic identities [13] (Fig. 1b). Uniparental embryos cannot develop to term because the contributions of maternal and paternal genomes, although necessary, are not represented equally [16–18]. There are more than 80 imprinted genes in humans and mice [19]. One key imprinted gene cluster affecting development is *Igf2-H19* [20], which is regulated by differentially methylated regions (DMRs). The epigenetic states of haESCs are inherited from the gametes. This guarantees their development during the intracytoplasmic injection processes [21]. However, the developmental ability of these haESCs diminishes significantly after long-term cell culture due to the loss of imprinting (such as *H19*-DMRs). By knocking out *H19*-DMRs, androgenic haESCs can stably retain their developmental potential [22, 23] thereby offering a promising option in the production of transgenic animals via intracytoplasmic injection (Fig. 1c). Based on this strategy, two groups separately converted the state of parthenogenic haESCs to the paternal epigenetic-like state using *H19*-DMR and *IG*-DMR knockout systems (Fig. 1c). Full-term offspring were then generated via intracytoplasmic injection of these modified parthenogenic haESCs [24, 25]. For primate species, haESCs exhibit typical characteristics of pluripotent stem cells [5], but more investigation is needed to determine whether primate haESCs have similar reproductive functions to rodent haESCs.

Medaka fish haESCs can maintain the haploid status in multiple phases of differentiation [2], while mammalian haploid cells undergo diploidization during the culturing and differentiation processes. This raises a question about the duration and ability of haESCs to maintain their haploid state during mammalian differentiation. Shuai et al. [26] illustrated that mouse haESCs retained their haploidy and pluripotent states in both epiblast and neuronally differentiated stem cell stages. Similar results were also found in human haESCs [7], indicating that mammalian haploid cells have great potential in various lineage-specific genetic screening applications [10]. Another type of mammalian haploid cells can be observed through the study of near-haploid human tumor cells. Long before the establishment of authentic human haESCs, near-haploid cell lines had been derived from leukemia and solid tumors [27]. This cell line showed great advantages in screening for drug resistance or disease-related genes. However, like other cancer cells and thus posing a potential risk in their application, near-haploid cells tolerate many copy-number variations in their genome.

### Instability of the mammalian haploid genome

**Fig. 2:**
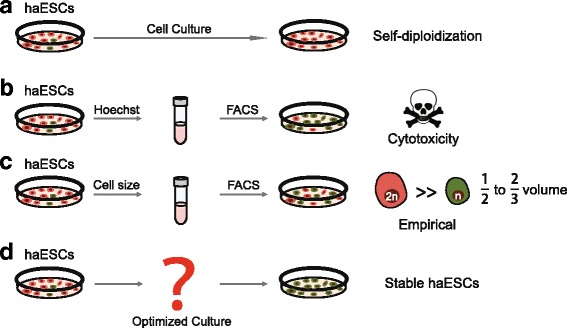
Optimizing haESC culture and enrichment systems. **a** During daily cell culture, haESCs undergo severe self-diploidization thus preventing their application in genetics. **b** Although haESC concentrations can be enriched using the Hoechst 33342 sorting method, the effect of Hoechst 33342 is toxic and does great harm to haESCs. **c** Since haploid ESCs are half to two-thirds the size of diploid ESCs, they can be sorted according to their physical parameters. However, this strategy is subjective and difficult to judge. **d** An optimized culturing system which allows for the perpetual maintenance of haESCs is needed. FACS fluorescence-activated cell sorting, haESC haploid embryonic stem cell

The derivation of mammalian haESCs failed almost 30 years ago due to genome instability. The phenomenon of diploidization was found during the first attempt at mouse haESC establishment [28]. The development of fluorescence-activated cell sorting (FACS) technology made it possible to enrich for haploid cells and helped to derive the first mouse haESCs [3]. Some of the haESCs would spontaneously diploidize in daily culture, so periodical FACS was indispensable for haploid cell enrichment (Fig. 2a). Mouse haESCs require more frequent sorting than primate haESCs; perhaps because mouse ESCs proliferate at a higher rate. However, the exact mechanism remains unclear. Generally, when performing FACS, haESCs are stained with Hoechst 33342 and sorted using a 355-nm ultraviolet (UV) laser. However, DNA-binding dyes and UV activation are harmful to the cell and affect the likelihood of survival (Fig. 2b). In order to achieve haploid purification, some groups have tried to develop an alternative method to DNA straining. Haploid ESCs are half to two-thirds the size of the diploid cells [29] meaning that the diameter of haESCs ranges from 10.0 to 15 μm at the G1 phase [30]. Taking advantage of physical differences in diameter and using diploid ESCs as controls, it is easy to distinguish the haploid cell population using forward scatter (FSC) and side scatter (SSC) on a scatter plot generated from sorting of these cells [31] (Fig. 2c). Although the enrichment of haESCs by cell size circumvents the toxicity due to the DNA dye, this method is subjective and requires experience to gait the haploid population precisely.

Although optimization of the FACS method is needed, describing the mechanism associated with self-diploidization is of greater importance (Fig. 2d). An accepted hypothesis is that abnormal cell cycle regulation causes cells to skip the M phase and re-enter the G1/S phase so as to duplicate DNA. Takahashi et al. [32] developed a culture medium using a small molecular inhibitor of the Wee1 kinase in order to accelerate the G2/M phase transition and prevent cells from re-entering the G1/S phase. In this culture medium, haploid cells could last at least 4 weeks without FACS, providing supportive evidence that cell cycle regulation may be a reason for diploidization. Recently, an interesting study showed that the duration of the metaphase in haESCs is significantly longer than that in diploid ESCs, while a chemically induced delay in mitosis strengthened the observation of diploidization [33]. Meanwhile, the consistent maintenance of haploidy in primates and humans is greater than that observed in rodents. While the hypothesis associated with this finding needs further validation, proliferation kinetics is thought to be relevant to self-diploidization. Nevertheless, the specific pathway(s) regulating diploidization has not yet been found. Since diploidization compromises the application capacity of haESCs, it is important that the genomes of haESCs are stabilized.

### Genetic screening

Genetic screening is the prominent application of haploid stem cell technology. *Saccharomyces cerevisiae* was used as a model organism to study haploid gene function before the derivation of haESCs [1]. However, due to species specificity, this approach cannot be directly applied to mammalian systems. Although the human genome project has successfully sequenced all 3 billion chemical units and identified approximately 20,000–25,000 genes in the human genome, their relevant functions are yet to be elucidated fully and require further study. Global genomic screening has therefore been widely used in mammalian ESCs in order to clarify gene function in many biological procedures. However, it is difficult to obtain homozygous mutations in diploid ESCs. Additionally, heterozygous genotypes may have no impact on their phenotype for the homologous allele complement. This means that diploid genomes hamper the study of recessive genetic conditions. Since haploid cells only carry one set of chromosomes, they exhibit corresponding phenotypes in the presence of a mutation. Genetic screening in mammalian cells often directly promotes medicinal and pharmacological research. Currently, it is difficult to obtain double homogeneous allele mutations through genome editing techniques. This hinders the generation of homozygous gene knockout libraries. The use of haESCs, however, can overcome this obstacle.

**Fig. 3:**
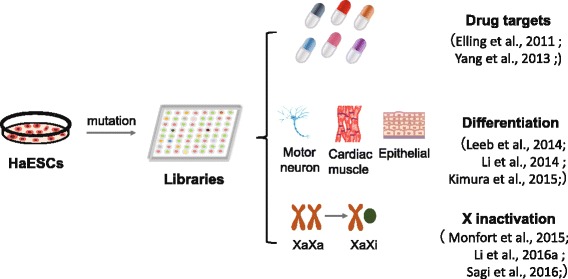
Application of haESCs in multiple types of genetic screening. Since haESCs contain a haploid copy of the genome, it is easy to obtain homozygous mutant libraries through transposon or viral systems. To date, mutant haESC libraries have been exploited in various targeted gene screening systems in order to track cell fate. Such systems include factors such as toxic resistance [4, 5], cell differentiation [6, 37, 48], and X-chromosome inactivation [7, 35, 36]. haESC haploid embryonic stem cell

Typically, genetic screening aims to obtain loss-of-function phenotypes through allele mutation. Genomic engineering is applied to produce mutant libraries through transposon-mediated insertion or nuclease-mediated targeting modification technologies—including *piggyBac*, Clustered regularly interspaced short palindromic repeats/Cas (CRISPR/Cas), and transcription activator-like (TAL) effector nucleases (TALENs) [34]. Once screened and tested, specific mutated cells are said to have been generated. Hitherto, through gene screening on haploid cell lines, basic studies including DNA repair, drug toxicity, X-chromosome inactivation, cell differentiation, and human clinical diseases have been investigated separately [3–5, 7, 8, 23, 29, 35–43] (Fig. 3). Primate haESCs can maintain haploidy in long-term culture, which makes them good resources for gene function research [5, 7, 8]. All of the established mammalian haploid cells, including near-haploid human tumor cells, have been applied in whole-genome genetic screening at the cellular or organism level, and many accomplishments have been achieved to date. Leeb et al. [37] found that Zfp706 and Pum1 were key regulators governing differentiation of naïve stem cells by screening mutated haESCs. Recent gene editing techniques facilitated the efficient utilization of haESC resources, such as CRISPR/Cas9 knockout libraries, to acquire offspring carrying multiple heterozygous mutations [23, 44]. In addition, gene trapping with a *piggyBac* transposon is a more efficient approach in the genetic screening of haESCs, because it allows more precise assessment of the integration site than when using CRISPR/Cas or TALENs [6, 45]. Taken together, gene screening in haESCs boosts the basic and clinical research fields of developmental biology and regenerative medicine [34]. This holds great value in the study of cancer, species evolution, biomolecular interactions, lineage specification, and signal pathways.

### A platform to study X-chromosome inactivation

Along with many genetic screening traits, haESCs are ideal tools to study X-chromosome inactivation (XCI). In diploid cells, the expression ratio of X chromosomes to autosomes (X:A) is 1:2, while the ratio of X:A is 1:1 in haploid cells [29]. XCI is a mammalian-specific, sophisticated process involved in epigenetic modifications and developmental processes. There are three types of XCI in mammals: XCI through imprinting, random XCI which happens in female development, and transcriptionally inactivated XCI during meiotic prophase I in males [46]. In mammals, dosage compensation occurs in XX females with the expression of the long noncoding X-inactivation-specific transcript (*Xist*) which randomly inactivates one of the two X chromosomes [47]. Overexpression of *Xist* can also initiate XCI.

Monfort et al. [35] used a dox-inducible *Xist* overexpression system to screen for key regulators in XCI. They identified a RNA-binding protein SPEN which is required for *Xist* RNA localization and recruitment of chromatin modifications. This system serves as a good example of using haploid ESCs when studying gene-silencing pathways and epigenetic modifications. Li et al. [36] generated mouse–rat allodiploid ESCs through fusion of haploid ESCs between the two species. They reported an interesting phenomenon in that the allopolyploid cell lines exhibited special gene expression patterns and mouse-specific XCI during differentiation. Furthermore, they systematically analyzed the bulk RNA-seq data, and found 146 unknown mouse genes potentially escaping XCI in the allodiploid differentiated somatic cells. Therefore, haESCs offer a brand new platform to study the XCI process in mammalian systems.

## Conclusions

Because of their pluripotent properties and the presence of a haploid genome, haESCs can be a powerful tool and a valuable resource in studying gene function. If haESCs could be cultured in a stable manner that prevents diploidization in the future, high-throughput mutated haESC lines would provide more opportunity to study mechanisms behind the development and presence of genetic disorders and susceptibility to diseases. Hence, haESCs can serve as a potentially versatile research tool in many fields, including dosage compensation, long noncoding RNA, XCI escape, and in-vitro meiosis. Based on the developmental potential of haESCs, transgenic animals are easily bred via intracytoplasmic injection. This provides a convenient way to produce numerous homozygous, mutated animals for the study of diseases caused by genetic mutation(s).

## Abbreviations

CRISPR/Cas: Clustered regularly interspaced short palindromic repeats/Cas
DMR: Differentially methylated region
ESC: Embryonic stem cell
FACS: Fluorescence-activated cell sorting
FSC: Forward scatter
haESC: Haploid embryonic stem cell
SSC: Side scatter
TALEN: Transcription activator-like (TAL) effector nuclease
UV: Ultraviolet
X:A: X chromosome to autosome
XCI: X-chromosome inactivation
*Xist*: X-inactivation-specific transcript
